# Facilitators and barriers to implementing successful exclusion among children with shiga toxin-producing *Escherichia coli*: a qualitative analysis of public health case management records

**DOI:** 10.1186/s12889-024-19580-w

**Published:** 2024-08-21

**Authors:** Amoolya Vusirikala, Yanshi, Charlotte Robin, Sam Rowell, Girija Dabke, Georgina Fox, Jade Bell, Rohini Manuel, Claire Jenkins, Nicola K Love, Noel McCarthy, Dana Sumilo, Sooria Balasegaram

**Affiliations:** 1 UK Health Security Agency, London, England, UK; 2 UK Field Epidemiology Training Programme, UK Health Security Agency, London, England, UK; 3 NIHR Health Protection Research Unit in Gastrointestinal Infections, Liverpool, England, UK; 4 School of Medicine, Trinity College Dublin, Dublin, Ireland; 5 Warwick Medical School, School of Medicine, Warwick, England, UK

**Keywords:** Shiga toxin-producing *Escherichia coli*, Childcare setting, Exclusion policies, Adherence, Qualitative analysis

## Abstract

**Background:**

Shiga toxin-producing *Escherichia coli* (STEC) infections are a significant public health concern as they can cause serious illness and outbreaks. In England, STEC incidence is highest among children and guidance recommends that children under six diagnosed with STEC are excluded from childcare until two consecutive stool cultures are negative. We aimed to describe the barriers and facilitators to implementing exclusion and the impact of exclusion policies on young children and their families.

**Methods:**

Individual level data was obtained from a wider study focusing on shedding duration among STEC cases aged < 6 years between March 2018 – March 2022. Data was extracted from England’s public health case management system. The case management system includes notes on telephone conversations, email correspondence and meeting minutes relating to the case. Collected data consisted of free text in three forms: (1) quotes from parents, either direct or indirect, (2) direct quotes from the case record by health protection practitioners or environmental health officers, and (3) summaries by the data collector after reviewing the entire case record. We analysed free text comments linked to 136 cases using thematic analysis with a framework approach.

**Results:**

The median age of included cases was 3 years (IQR 1.5-5), with males accounting for 49%. Nine key themes were identified. Five themes focused on barriers to managing exclusion, including (i) financial losses, (ii) challenges with communication, engagement and collaboration, (iii) issues with sampling, processing, and results, (iv) adverse impact on children and their families and (v) conflicting exclusion advice. Four themes related to facilitators to exclusion, including (i) good communication with parents and childcare settings, (ii) support with childcare, (iii) improvements to sampling, testing, and reporting of results, and (iv) provision of supervised control measures.

**Conclusions:**

Qualitative analysis of public health case records can provide evidence-based insights around complex health protection issues to inform public health guidelines. Our analysis highlights the importance of considering wider social and economic consequences of exclusion when developing policies and practices for the management of STEC in children.

**Supplementary Information:**

The online version contains supplementary material available at 10.1186/s12889-024-19580-w.

## Background

Shiga toxin-producing *Escherichia coli* (STEC) belong to a pathogenic group of *E. coli*. STEC infections pose a significant public health threat due to the potential severity of disease. Symptoms can range from mild diarrhoea to haemolytic uraemic syndrome (HUS), which is the most common cause of acute renal failure in children, and can be fatal [1]. STEC is diagnosed by laboratory testing of the stools of an infected person. STEC are zoonotic and spread from animals to humans via direct contact or consumption of food or water contaminated with animal faeces or can spread from person-to-person by the faecal-oral route. The infectious dose is low, facilitating transmission and increasing the potential to cause large outbreaks [2].

Outbreaks of STEC infection occur in childcare settings and this may be due to a combination of factors including prolonged shedding, poor/under-developed personal hygiene measures and immature immune systems in this age group [3]. To limit transmission, public health authorities often require children to stay away from childcare settings until laboratory tests confirm they are microbiologically clear of STEC bacteria [2, 4, 5]. In England, children under the age of six are excluded from childcare settings, until they have two consecutive culture-negative stool samples [2]. Prolonged carriage in children can result in lengthy exclusion [6, 7]. Current exclusion policies can result in disruption to families in terms of potential loss of earnings, the child’s education, emotional and mental stress and disengagement with the health system [8].

In England, it is a legal requirement that all cases of STEC are reported to the national public health institute, the UK Health Security Agency (UKHSA) [9]. Regional health protection teams (HPTs) within UKHSA record each case on a national public health case management system and public health follow up is often managed jointly by HPTs and Environmental Health Officers (EHOs) in local authorities [2].

A previous study in England identified challenges in implementing exclusion in 30% of children with STEC [6]. The study identified parental anxiety and/or communication issues as the most frequently reported challenges encountered, followed by concerns over social isolation, disruption to family life, sampling issues, financial hardship and childcare. However, this study did not contribute any further insights beyond stating the encountered difficulties. Given the accumulative evidence of socioeconomic inequalities in gastrointestinal infections [10, 11], it is becoming increasingly important to explore the hardships experienced by families and consider how these factors may play a role in adherence to exclusion policies. There is scarce research on parental experiences of prolonged exclusion due to STEC and understanding their perspective is important to facilitate better engagement with public health authorities.

To our knowledge, this is the first study to qualitatively assess the barriers and facilitators to STEC exclusion by analysing public health case record data. By doing so, this paper aims to fill the existing research gap and enhance our understanding of factors influencing successful exclusion. These insights will inform recommendations to guide effective case management, promote carers’ engagement with public health policies and facilitate compliance.

## Methods

### Data source

Data was obtained as part of a suite of studies undertaken among STEC cases aged < 6 years attending childcare settings with an onset date or sample date from 31st March 2018 and 30th March 2022 [12].

Data was collected from the national case management system used by HPTs to manage cases of infectious disease including STEC. The case management system includes notes on telephone conversations, email correspondence and meeting minutes relating to the case. Public Health England was the predecessor of UKHSA and as the study data collection period encompasses the times when both PHE and UKHSA served as the national public health institute, both organisation names are referenced.

The present study focuses on data regarding challenges with managing exclusion and measures to help manage exclusion.

### Data collection

This qualitative document review involved data extraction performed by trained health protection practitioners (*n* = 3) or members of the national gastrointestinal team (*n* = 2). Following review of the case record, relevant data was extracted into an electronic data collection tool produced in Snap Survey (Snap 11 Professional), a web-based survey tool. Case records that included free text information on barriers to, or facilitators of, exclusion in the data collection tool, were included in this study. Collected data consisted of free text in three forms:

1. Direct or indirect quotes from parents (Parents reported). Indirect quotes from parents are defined as direct quotes from the case record which have been written by the health protection practitioner that directly spoke to the parents of the case e.g., “Called mother, she said she is frustrated” whereas a direct quote from parents would be a quote from an email from the parent in the case record.
2. Direct quotes from the case record (HPT/Environmental Health Officer (EHO) reported).
3. Summary created by the data collector following review of the entire case record (Reviewer noted). This format may have been recorded by the data extractors in cases where extensive notes were present in the case records.

The data source is the operational record of the public health agency, which may reflect the perspective of the practitioner creating the record but also include some direct quotes from parents.

### Analysis

We used thematic analysis with a framework approach to analyse the data [13]. Data from the survey was imported into Microsoft Excel and was anonymised prior to analysis. Familiarisation with the data was achieved by reading the free text several times by two authors. Initial coding was performed on approximately 20% of the data by two authors independently. The coding framework was developed based on both a priori themes arising from previous research (e.g., parental anxiety, communication issues, disruption to family, social isolation, financial issues, childcare issues and sampling issues), and new themes identified from the initial coding of the data [6]. Consensus on a coding framework was reached through discussion with the study team. This framework was then applied to the remaining free text comments by one author and used to capture key themes of interest. The coding framework was applied inductively, to allow new codes and themes to be added as analysis progressed. The themes were added and modified until data saturation was achieved.

## Results

### Characteristics

Between 31st March 2018 and 30th March 2022, 367 cases of STEC aged < 6 years old with a known serotype had evidence of attending a childcare setting [12], of which 37% (*n* = 136) included free text information on barriers to, or facilitators of, exclusion in the data collection tool and were included in the study. The median age of included cases was 3 years (IQR 1.5-5), and males accounted for 49%; these were representative of the wider study population. Of the cases with ethnicity information (*n* = 104), 81 (77%) were White, 11 (11%) were Mixed or Other, 8 (8%) Asian and 4 (4%) Black. Clinical presentation of this subset of cases was comparable to the wider study population with diarrhoea or bloody diarrhoea reported in 95% and 43% of cases, respectively, and 8% of cases with HUS. There were 14 (10%) asymptomatic cases.

### Overall themes

We identified nine key themes. Five themes focused on barriers to managing exclusion, including (i) financial losses, (ii) challenges with communication, engagement and collaboration, (iii) sampling, processing, and results, (iv) adverse impact on children and their families and (v) conflicting exclusion advice. The other four themes related to facilitators to exclusion, including (i) good communication with parents and childcare settings, (ii) support with childcare, (iii) improvements to sampling, testing, and reporting of results, and (iv) provision of supervised control measures. An overview of all themes identified, along with additional free text comments that informed the framework and classification of the themes is provided in Supplementary Table S1 (Table S1).

## Key barriers in managing exclusion

### Financial losses

The financial losses incurred by the family due to exclusion were one of the major challenges encountered. Several cases had working parents, who had to take extended unpaid leave during the prolonged exclusion period, while still paying for the nursery fees.

The parents have been paying full nursery fees for 5 weeks now and having to take unpaid leave because the child is still not able to go back to nursery because of lack of results. This is costing them around £1500 a week [mum got very upset at this point]. (Reviewer noted).

I cannot financially have any more time off work[, ] nor can I afford to keep paying for nursery fees he isn’t utilising. (Parents reported)

Parents also felt pressure from their employer to return to work.

The mother is very anxious to get back to work as she is not getting paid. Mum is unable to go to work whilst child is at home awaiting clearance. She is ringing EHO multiple times a day in tears as she has received a warning from work. (Reviewer noted)

Additionally, parents who were self-employed could not work properly and had to bear financial losses.

Exclusion of child prevents his mother from working. She is self-employed so losing out financially. (Reviewer noted)

Several queried if financial compensation was available to support families with nursery fees and living expenses due to lost income.

Mother asking if there is any financial support as she is still needing to pay nursery fees. (Reviewer noted)

### Adverse impact on children and their families

Parents were particularly concerned about the loss of education, and the physical, mental and social impact the duration of exclusion would have on their child.

Case has been out of nursery for over a month ……. Mother also concerned of the impact this is having on the case with him being out of nursery for so long and not being able to have contact/socialise with other children[, ] this is beginning to impact him. (Reviewer noted)

A very frustrated, nearly 3[-]year[-]old who is at home and not in her usual routine of attending her education. This is going to really unsettle [child] emotionally when she does have to return and then settle back into her routine. (Parents reported)

Families also reported to have experienced stress, disruption and health issues due to exclusion of their children.

The stress of the situation was causing stress on mother’s marriage and causing her physical and mental health problems. (Reviewer noted)

Mother is asking for any advice as the case is asymptomatic but still testing positive, and it is becoming disruptive to the lives of child and mother. (Reviewer noted)

Furthermore, parents of children with special educational needs and disabilities (SEND) emphasised exclusion intensified an already complex situation, burdening the entire family.

Case is autistic and she is on reduced school hours due to high anxiety, it has been difficult settling her into school so the more time off the harder it will be for her to go back. Case has complex behavioural issues and staying at home is putting a lot of strain on the family. (Reviewer reported)

Additionally, some cases had limited family support, with no other alternatives for childcare which further caused difficulties in managing the exclusion.

Mum is very understanding of the situation around why we need to get clearance but is struggling with childcare provision as she normally works: she said it is very tight for her at the moment and that she has used up all her leave, and does not have family around. Difficulty finding alternat[ive]. (Reviewer noted)

### Challenges with communication, engagement and collaboration

Analysis highlighted that there was lack of parental engagement with the authorities. For example, some parents did not submit samples, or respond to calls.

Case’s family were extremely hard to communicate with so there were periods of non-response to messages, calls etc. and no clearance samples being submitted. (Reviewer noted)

In some instances, parents were *“disgruntled”* with the clearance process. Some parents questioned the exclusion guidance, particularly concerning the reasons for exclusion when a child was asymptomatic and the requirement for two negative samples.

Mum felt that HPT didn’t understand the situation that she was in having a healthy child at home, trying to work fulltime and paying nursery fees. Nursery had all the PPE [personal protective equipment] in place and child was having a solid stool, she had never been symptomatic, she didn’t understand why she could not take [child] into nursery. (Reviewer noted)

Mother struggling with childcare during prolonged exclusion and questioned why the child could not return as they were symptom free. (Reviewer noted)

At other times, the parents were frustrated with the process of exclusion and case management and ignored the exclusion advice from EHO.

Mum expressed that she was very frustrated by how this situation has been handled by GP/EH[O] as she felt she had been given conflicting advice and the way she had been notified of her child’s diagnosis at the beginning was very unprofessional. (Reviewer noted)

EHO informed parents that child needs to be excluded but, despite this they sent the child into nursery anyway. (Reviewer noted)

Analysis also highlighted the challenges HPTs faced while engaging with parents who were seeking their own information from other sources.

Father had been googling and was arguing that exclusion not necessary. (Reviewer noted)

In other cases, the parents preferred to engage with their GPs or testing at private labs.

Family did not want to use PHE clearance process and used GP surgery for clearance sample as more convenient for them. (Reviewer noted)

Parents preferred to submit samples to GP surgery and not via our [postal sampling] system (HPT reported).

In a specific case, the family had travelled abroad in the middle of the case’s clearance process and submitted a sample there, which was negative for *E.coli*. The reviewer also noted this resulted in the family being quite resistant and distrustful towards the UK process.

Some parents were not aware of exclusion or misunderstood the advice given to them, which resulted in children returning to childcare settings before the exclusion period was over.

Mother assumed the results from the second clearance sample was negative and so sent child back to school before being informed of the result.” (Reviewer noted)

Parents misunderstood exclusion advice and child went back to school without any negative clearance samples. (Reviewer noted)

In certain cases, language barriers were the key factor in the misunderstanding of advice provided by public health teams.

Some language barriers resulting in confusion with sample/form labelling.” (Reviewer noted)

Language barrier, family did not understand clearance, child went back once symptom free for 48 h. (Reviewer noted)

### Issues with sampling, processing and results

Several issues causing delays in the sampling process were reported by parents and HPTs.

Samples submitted to GP surgery but not logged so potentially lost. (HPT reported).

This [Case being out of nursery for over a month] has also not been helped by the hospital losing 2 of his stool samples and the most recent sample being “delayed” due to not being tested correctly. (Parents reported).

Further, the length of time for processing the samples, and notification of the results was seen to be prolonged, which disappointed many parents.

Called an unhappy Mum to say [reference laboratory] reporting sample for [date 1] and [date 2] are still showing as Pending. Will check again tomorrow and report asap. She complained this is “ridiculously slow”. (HPT/EHO reported)

Mother very frustrated at length of time it takes to get results of clearance samples. Mum says they are submitting 4–5 samples per week but haven’t had any results now for 11–12 days. (Parents reported)

Moreover, in some cases incorrect and contradictory clearance results were provided, which further caused frustration among parents.

[Date 1] – Mum received a text this morning saying case was positive and another one this evening saying she was negative. Has also received another text which says still positive but has no name and may be about case’s sister. Mum is not happy about the confusing text messages she has receive[d]. (Reviewer noted)

### Conflicting exclusion advice

In some cases, conflicting exclusion advice was provided by the different authorities, including clinicians, hospitals, GPs and EHOs.

She [Mother] was incorrectly advised by the GP to delay exclusion sampling for a week. HPT later corrected this advice” (Reviewer noted)

Mother states EHO discussed child returned after 1 negative sample so was very angry to learn from HPT it was in fact 2. (Reviewer noted)

Some other cases indicated multiple agencies gave conflicting advice, which confused the parents.

Conflicting information from GP/hospital clinicians & HPT on diagnosis, sample results and exclusion requirements received by parents. Were initially told by hospital that case did not have E. coli and was clear to return to nursery - at this stage diagnostic sample results were still positive. (Reviewer noted)

On one occasion, the GPs were unsure of exclusion advice, and required HPT support.

[GP] spoke to the mother of the patient she is concerned with advice PHE have given - she has been told to keep child off nursery even though she is asymptomatic. Mother cannot work if child is not able to attend nursery. So would like clear guidance issued by PHE. GP would like PHE to call (Reviewer noted)

On some occasions, the conflicting advice given by the GPs/EHO/hospitals resulted in cases returning to their settings before achieving clearance.

Mum of case claimed to have received a call to say that samples were negative, and case could return to nursery. (Reviewer noted)

Family advised by A&E that [case] could return to setting prior to exclusion advice being provided from HPT/EH[O]. (Reviewer noted)

## Facilitators to manage exclusion

### Good communication with parents and childcare settings

When consistent and comprehensive communication between UKHSA and parents was maintained, this helped parents to manage difficulties that arose when their children were excluded from school or nursery:

Frequent and regular communication to parents assisted with keeping them on board. In addition, a proper explanation of the rationale including legal remit & evidence base behind the request did actually help to calm the parents (Reviewer noted)

There were instances that indicated that the parents wanted to be regularly updated.

Mum is very keen to be kept in the loop about results/to have as much communication as possible. (Reviewer noted)

The HPT were able to provide detailed information to parents on the risk of STEC infection to others, the necessity of exclusion, sampling processes and possible timescales, the legal basis for exclusion, and hygiene precaution arrangements.

HPT communicated regularly with family to explain a complex and frustrating process. (Reviewer noted)

Parents were frequently kept informed about each step of the process through phone and email to avoid confusion that came with the involvement of multiple agencies in the exclusion process:

Due to complexities with the clearance arrangements & involvement of various stakeholders, parents were starting to get confused as dealing with so many different people. HPT outlined clearly in an email a response to parents’ concerns and questions to provide some clarity including a plan for return to school (Reviewer noted)

[Date] – I have this afternoon spoken to the mother of [child] and explained the process, the result and the results to come; and promised to let her know the outcome tomorrow afternoon. I have asked her to keep going with the samples till we say stop. [Date] – Called [child] Mum and gave her the good news that [child] can at last return to nursery almost 3 months after [child] original onset. Very happy Mum! (HPT/EHO reported)

EHOs developed good working relationships with parents. It helped the parents to overcome issues encountered during the exclusion process and ensure compliance with exclusion requirements.

EHO formed good working relationship with parents which helped to smooth out the issues. (Reviewer noted)

EHOs developed good relationship with mother especially which helped to ensure compliance with exclusion requirements. (Reviewer noted)

Schools were also regularly contacted to give updates and multi-agency meetings were held when needed.

Joint meeting with parents and HPT and UKHSA national team and school visit to school with HPT, CICN [Community Infection Control Nurse], EHO, headteacher. (Reviewer noted)

### Support with childcare

The analysis also highlighted that support with childcare was an important facilitator in parents’ ability to adhere with exclusion advice. In some cases, the HPT and EHO communicated with parents’ employers, explaining the need for the parents to have time off work due to exclusion of the child from childcare settings.

HPT wrote to employer requesting they allow family to have paid carer’s leave. (Reviewer noted)

Email sent to headteacher of parent (mum) by EHO explaining the need for parent to have time off work as child cannot be sent to childcare setting until two clearance samples are received. (Reviewer noted)

In other settings, it was not needed as childcare was provided by grandparents or family friends. On certain occasions, childcare providers were requested by the HPT team to waive fees. HPTs also advised the parents to reach out to local authorities and Citizens Advice for financial support.

HPT advised mother to seek advice from the LA [local authority] regarding an emergency payment if available to support compliance. Otherwise, to contact Citizens Advice for further benefits information (Reviewer noted)

### Improvements to sampling, testing, and reporting of results

HPTs and EHOs facilitated timely submission and testing of samples, as well as prompt reporting and communication of sample results to the relevant authorities, which helped to manage the exclusion. Measures taken included HPTs opting for couriers to transport samples instead of GP sampling and EHOs personally assisting with sampling at home.

HPT tried to speed up testing process by communicating directly with the lab and arranging with EHO and lab for results to be reported over the weekend. (Reviewer noted)

EHO visited case’s home to take samples themselves to ensure no further confusion. (Reviewer noted)

HPT were honest about the mistakes at the laboratory and investigated the issues with the laboratory directly to obtain clarification and latest clearance results:

Email sent to parent by consultant: …. I apologise for the inconvenience you have faced in this process …. I am sorry that the clearance sampling was difficult for [Name]. The process is managed by multiple organisations and appreciate that it could be improved. I have spoken with colleagues and will be reviewing to try and make sure the delays are less likely to be repeated in future. (HPT/EHO reported)

They continued to regularly check for the results and liaised with the GP to ensure parents receive the results from EHO or HPT as soon as possible:

I will continue to chase results and before too long we will see the infection clear – they always do! (HPT/EHO reported)

### Provision of supervised control measures

In some cases, a risk assessment was conducted by HPTs, EHOs, infection control nurses, childcare setting and parents, and children were allowed to return to the setting before achieving clearance, with extra hygiene and handwashing controls in place.

EHO observed hand washing – was assessed as thorough and competent. EHO also visited the school and confirmed with the inclusion teacher that case would be supervised with hand washing after using the toilet and before meals until she had two negative stool samples. (Reviewer noted)

## Discussion

This qualitative analysis of public health case records enhances our understanding of barriers and facilitators in managing exclusion for children with STEC. The study’s findings emphasise the complexity of exclusion, which presents challenges for a range of stakeholders, including children, parents, various public health authorities, parents’ employers and childcare organisations. There are considerable social, emotional and economic burdens placed on families that need to be considered when implementing exclusion policies.

### Barriers

A comprehensive understanding of barriers to exclusion in the context of STEC is vital. There may be similarities in the barriers identified for compliance with, for example, COVID-19 self-isolation [14–16] but the potentially lengthy exclusion period for STEC may amplify these barriers. Our findings highlight the financial and social consequences of exclusion policies on families. Working parents caring for excluded children experience both loss of income from being out of work for a prolonged period and ongoing childcare expenses. Previous research around COVID-19 often identified lack of financial support for people self-isolating as a key factor in not engaging with public health interventions [15–18]. With higher rates of GI infections in children from more disadvantaged groups [10], the financial costs of exclusion policies can have more severe economic consequences for these groups and widen already existing inequalities. The emotional strain of exclusion policies on both the carer and children is evident from our analysis. Parents’ concerns for their children were predominately around disruption to usual routine and reduced social and physical contact with others. These effects have been shown to have a negative impact on the psychological and physical wellbeing of children in terms of anxiety, sleep and appetite, particularly in young children with SEND [19]. The emotional and physical toll on parents identified may be attributed to balancing attempting to comply with the guidelines while doing what is felt best for their child [20]. For working parents, we also identified the pressure from their employers to return to work as a contributing factor to distress experienced in the exclusion process.

Our study also identified that challenges in cooperation with public health authorities in the clearance process were key barriers. This was often attributed to a lack of understanding of the ongoing risk of transmission in this vulnerable population after symptom resolution, the purpose of clearance samples and how these aspects fit with the overall exclusion guidance. Our analyses indicated that some parents believed measures were unnecessary for asymptomatic children, a rationale that was often cited as a key belief during the COVID-19 pandemic [21–23]. Previous research indicates that if carers perceive a threat as minimal, there is a risk that their child will return to the community while infectious [20]. A lack of clarity on the rationale of preventative health measures contributed to the confusion around advice on COVID-19 provided and ultimately poorer adherence [24]. Moreover, it became evident that there were difficulties encountered in sample processing, and while our study did not uncover specific reasons for these challenges, anecdotal evidence points toward incomplete sample forms or mislabelling of samples playing a role, indicating a possible need for improved clarity in the sample submission process for parents.

Our findings also suggest that disengagement with HPTs could be due to parents’ preference of completing the clearance process through their GP which may reflect the higher level of trust patients place in familiar medical professionals.

With families already having to navigate dealing with multiple authorities (HPTs, EHOs, GPs and hospitals), contradictory messages from these parties added further to frustration and sometimes resulted in children returning early to childcare setting. Lack of awareness of guidelines among clinicians outside of public health agencies may account for some of the variability in advice. Studies have shown receiving inconsistent information can lead to increased confusion, stress and loss of confidence in authorities [20, 25, 26].

Financial concerns and concerns about the impact on the child’s education and well-being already pose challenges to compliance with exclusion. However, the addition of having a child who is asymptomatic or experiencing delays in receiving clearance results can make an already challenging situation far more difficult and amplify existing challenges. These issues then become more embedded barriers to engaging with HPTs. Recent implementation of new rapid testing methods and more efficient logistics for processing clearance samples in laboratories local to the case should improve the timeliness of reporting results back to the parents.

### Facilitators

Our analysis not only highlighted the importance of regular, honest and detailed communication between public health teams and parents as a key facilitator in effectively implementing exclusion but also communication between public health teams and all relevant stakeholders, including parents’ employers, childcare settings, GPs and laboratories. This supports previous research which has shown open communication outlining reasons for recommended actions increases perceived credibility of authorities and enhances compliance [27, 28].

Additionally, we also show that having one team take charge of liaising with all partners proves beneficial in expediting the clearance process, as well as potentially alleviating parents’ wide-ranging concerns. Facilitators identified in relation to testing were use of couriers and assistance with sampling at home in certain cases; this may not always be practical or cost-effective but is in line with prior research indicating speed and convenience of testing [18, 29] influence testing uptake. HPTs conducting regular risk assessments with partners resulting in implementation of precautions in settings enabled early return. Deploying this approach is dependent on the risk assessment and may only be appropriate for those children deemed as being a lower risk to return. Regular joint reviews of risk may demonstrate public health agencies’ proactive efforts to minimise the exclusion period for children, leading to improved relationships with families, ultimately enabling better adherence to exclusion policies. However, not all children can have early return; therefore, regular review may falsely raise parental expectation and cause more disappointment and thus may not be applicable for all cases.

#### Recommendations

The insights generated from our study highlight the importance of considering the wider social and economic effects of exclusion policies. Financial assistance programmes for lower income households are required to reduce widening already existing inequalities of GI infections.

Our study also identified examples of good practice. We suggest developing a toolkit for public health teams (HPTs in England) to support incorporation of this good practice into routine public health practice. The toolkit could be co-produced with families, childcare settings and local public health teams and be tailored for local use. It should include both existing and additional material such as frequently asked or inclusion questions (FAQs) and infographics for families and childcare settings, template letters for settings, employers and GPs, as well as a risk assessment checklist including questions on mental health and financial impact on children and families.

Aspects to be covered include:

1. Engage in detailed communication with parents, specifically emphasising the risk of STEC infection to others, the role of clearance in reducing asymptomatic transmission and the potentially lengthy clearance process.
2. Have access to translators who have previous experience of working in health protection.
3. Be aware of avenues of financial support locally, so practitioners have the ability to signpost families when needed.
4. Explain the situation to parents’ employers, which may add legitimacy to parents’ request for time off work or flexible working arrangements and alleviate tension between parties.
5. Liaise with childcare settings to promote the provision of support for affected children that may help with the detrimental social, mental and educational impact of exclusion.
6. Incorporate a holistic approach to regular risk assessments for children with prolonged shedding in close collaboration with parents, EHOs, public health microbiologists and childcare settings.
7. Raise awareness of STEC exclusion advice and the clearance sampling and reporting process among clinicians, to reduce the mixed messages parents receive.

### Strengths & limitations

This study represents the first effort to understand implementing STEC exclusion policy through qualitative analysis of public health case records. Unlike other qualitative studies that recruit participants after the event, public health case records offer information in real-time allowing us to analyse conversations that families were having at the point of child’s diagnosis and exclusion. This may provide a better reflection of experiences in terms of minimal recall bias. Furthermore, in the absence of participant recruitment, this method mitigates potential selection bias originating from families with specific experiences seeking participation in the study. However, this approach relies on secondary data, which was not originally collected for the specific purpose of this review and data may be incomplete and lacking standardisation. A key limitation of the study was that the free text responses that were completed after reviewing the case records did not always provide a comprehensive picture. For instance, the distinction between lack of engagement from the beginning and deliberate disengagement with the clearance process among carers could not be accurately discerned. Additionally, while having 20% of the quotes reviewed by a second researcher is pragmatic, reviewing all data by more than one author would have increased reliability. Another limitation to consider is that our study focused on cases where exclusion issues were reported. This subset represents only a portion of all cases, and it is possible that the remaining cases did not experience any problems with exclusion, which could indicate the effectiveness of the current system. While ethnography or interviews may offer deeper insights, these methods are more resource-intensive and expensive, with reported challenges in recruitment [30]. Using the public health case management system as a data source generates valuable insights into the complexities of managing exclusions, with the potential for greater generalisability at less cost, and can also generate hypotheses for subsequent in-depth qualitative studies.

## Conclusions

Qualitative analysis of public health case records is a pragmatic method to gather insights on the impact of exclusion policies on children and families. Effective implementation of childcare exclusion policies relies on developing public health practices that address the barriers and facilitators highlighted in this study. This approach can interrupt disease transmission while minimising social and economic consequences. We hope our findings inform more sensitive exclusion policies that ensure the public health benefit of exclusion is balanced against potential harm from exclusion.

### Electronic supplementary material

Below is the link to the electronic supplementary material.

Supplementary Material 1

## Data Availability

Data are incorporated into the article and material contained within. Individual level data cannot be shared due to ethical/privacy reasons. The datasets generated and/or analysed during the current study are not publicly available as the dataset is a part of clinical management database of the UK Health Security Agency, which is not publicly shared but are available from the corresponding author on reasonable request.
