# Supplementary material for: Facilitators and barriers to implementing successful exclusion among children with shiga toxin-producing Escherichia coli: a qualitative analysis of public health case management records
Source: BMC Public Health. 2024 Aug 21;24:2272. doi: 10.1186/s12889-024-19580-w (PMC11340135; doi:10.1186/s12889-024-19580-w)
Supplement: Supplementary file 1 — Supplementary Material 1 [file 12889_2024_19580_MOESM1_ESM.docx]

**SUPPLEMENTARY MATERIAL**

**Table S1. Overview of themes with selected additional examples of quotes in the themes**

|  | **THEME** | **OVERVIEW** | **QUOTE** |
| --- | --- | --- | --- |
| **BARRIERS** | Financial Losses | Parents did not want to exclude their child as otherwise they had to bear financial losses due to unpaid leave from work and unnecessary payment of nursery fees during the exclusion period. | *“Case has been out of nursery for over a month with mother having to take a month unpaid time whilst still paying for his nursery fees. (Reviewer noted) “This can no longer continue as this has left us in financial dire straits.” (Parents reported)*  *“Mum says she feels left in the dark and is concerned about having to have a prolonged amount of time off work.” (Parents reported)*  *“Mum was very understanding of having to keep child off nursery to prevent transmission, but her only concern is that she will have to take time off work to facilitate this and she will have to continue to pay the nursery anyway.” (Reviewer noted)*  *“Mother is finding it difficult to keep case at home as her work are demanding her to return but she has no childcare for the case if he can't go to nursery.” (Reviewer noted)*  *“Parents agreed to exclusion but was concerned over the possible duration as both parents work.” (Reviewer noted)*  *“Mum unhappy about exclusion due to important project at work and potential loss of earning. She is very agitated and anxious that the children need to be off school until the clearance samples are returned (Reviewer noted) “ Says she and her husband cannot afford more childcare, and cannot take more time off work, she says she will [lose] her job if she takes time off, that she must go into work tomorrow.” (Parents reported).*  *'“I cannot financially have any more time off work nor can I afford to keep paying for nursery fees he isn’t utilising.’’ (Parents reported)* |
|  | Challenges with communication, engagement and collaboration | Some parents did not want to engage in the exclusion process as they were unaware or not confident about the different aspects of the exclusion policy, such as the need for asymptomatic case’s exclusion, two negative samples, etc. | *“Parents were not happy about having to submit a further sample after first negative as couldn't see the point.” (Reviewer noted)*  *“Concerns around whether parents would comply with exclusion requests as case asymptomatic and parents refusing to exclude.” (Reviewer noted)*  *“Case's family travelled abroad in middle of clearance process. Whilst abroad had stool sample submitted in [country outside of UK] which was 'negative' for E. coli allegedly - this resulted in family being quite resistant and distrustful of UK process.” (Reviewer noted)*  *“Family did not respond to EHO or engage in clearance process initially.” (Reviewer noted)*  *“Mother read the national guidance herself – submitted two samples and decided to send the child back to nursery without knowing the results. She did this without discussing it with EHO or HPT.” (EHO/HPT reported)*  *“Parents have been "disgruntled" with regards to clearance.” (Reviewer noted)* |
|  | Issues with sampling, processing and results | Parents expressed their disappointment towards the long duration of sample processing and result notification. In some cases, parents shared feeling of frustration and anger due to incorrect results notification. | *“Parents frustrated as they were provided with incorrect results for a clearance sample in error.” (Reviewer noted)*  *“Initially GP sur[g]ery were informing mum that clearance samples were negative when they weren't as the surgery had not [r]eceived ref lab results for clearance samples and only had a result for a sample taken some time prior to the initial positive result.” (Reviewer noted)* |
|  | Adverse impact on children and their families | Parents expressed their concerns over the negative physical, social and emotional impact exclusion is going to have on their children and other family members, especially for children with special educational needs and disabilities. | *“Mum is struggling with anxiety - child hasn't been able to get back to nursery and she is concerned about his physical and developmental needs.” (Reviewer noted)*  *“The problem of the unpredictability of when he* (case) *might be clear has also meant she* (mother) *is frustrated and terribly upset.” (Reviewer noted)*  *“EHO reports family fed up with keeping child in. Mother is struggling to manage the situation at home as the case is autistic and needs routine and is unable to return to school at present. Mother runs business from home and exclusion of child is having an impact in her business.” (Reviewer noted)* |
|  | Conflicting exclusion advice | In some instances parents were given incorrect or contradictory exclusion advice by different agencies, which resulted in parents feeling confused. | *“I don’t think the impact of this is being taken into account considering the fact that [child] attended nursery on the advice of the GP, Public Health AND Nursery were all in agreement that she could still attend whist she was having the symptoms in the beginning.” (Parents reported)* |
| **FACILITATORS** | Good communication with parents and childcare settings | Where parents experienced regular communication from the public health authorities during the exclusion period of their children. | *“Parents kept constantly informed with progress of clearance samples and results.” (Reviewer reported)*  *“HPT took the time to contact parent to explain in detail the situation and what's required.” (Reviewer reported)*  *“Regular updates to family from HPT and also liaison with lab for results.” (Reviewer reported)*  *“Appears that constant updates to family and also liaison with lab assisted with the process.” (Reviewer reported)*  *“HPT spoke to mother very frequently to provide updates.” (Reviewer reported)* |
|  | Support with childcare | Actions taken by the HPTs and EHOs to support parents for childcare during the exclusion period supported adherence. | *“HPT called childminder 1 who: "is very happy to support mum and not charge and save place until end of feb as agreed - was very understanding and appreciated the difficult to mum - not in a hurry to replace his place as already has sister there - thanked her for being understanding and agreeing” (HPT reported)*  *“The mother had also been getting assistance from family members and friends to look after child who remained asymptomatic. EHO provided letter for mother of the case to show to her employer explaining why she was taking time off.” (Reviewer noted)* |
|  | Improvements to sampling, testing and reporting of results | Measures taken by the public health agencies to facilitate timely sample submission, testing, and early reporting of results, helped to decrease the exclusion duration. | *“EH[O] liaison with GP surgery to ensure mum only obtained clearance sample results from EH[O] or HPT.” (Reviewer noted)*  *“HPT investigated issue with lab directly and were able to obtain clarification and latest clearance results.” (Reviewer noted)*  *“HPT tried to ensure clearance samples were submitted, transported and tested as quickly as possible.” (Reviewer noted)*  *“HPT tried to ensure timely submission and testing of samples and regularly communicated with parents to keep them up to date on results.” (Reviewer noted)* |
|  | Provision of supervised control measures | Supervised control measures that were put in place in the childcare settings to reduce the exclusion period of the child at home. | *“Child was thought to have prolonged carriage - 7 positive clearance samples over the course of a month although child was symptom free. Multiagency meeting led by HPT - agreed that case could return to nursery (on [Date]) prior to clearance with risk assessed enhanced controls in place.” (HPT reported)* |
